# Factors affecting food security and poverty analysis among rural households in the North-Eastern Highlands of Ethiopia

**DOI:** 10.29219/fnr.v69.12014

**Published:** 2025-11-10

**Authors:** Umar Demisse Mohammed, Arega Bazezew Berlie

**Affiliations:** 1 Department of Geography and Environmental Studies, College of Social Science and Humanities, Woldia University, Woldia, Ethiopia; 2 Department of Geography and Environmental Studies, Faculty of Social Science, Bahir Dar University, Bahir Dar, Ethiopia

**Keywords:** households, factors, incidence, depth, severity, food security, Ethiopia

## Abstract

**Background:**

Ethiopia frequently experiences food insecurity due to recurrent droughts, food shortages, and famine. Understanding the determinants of food security and the extent of poverty is crucial for effective intervention.

**Objectives:**

This study aimed to identify key factors influencing food security and analyze poverty levels among rural households in the North-Eastern Highlands of Ethiopia.

**Methods:**

A mixed-methods research design was employed, utilizing both probability and non-probability sampling techniques to select 382 households, focus group discussants, and key informants. Quantitative data on food security and poverty indicators were analyzed using binary logistic regression (for determinants) and the Foster–Greer–Thorbecke (FGT) model (for poverty measurement).

**Results:**

The FGT analysis indicated that the incidence, depth, and severity of food insecurity were 55, 18, and 8%, respectively. The *Belg* (spring) livelihood zone and female-headed households exhibited higher food insecurity, while the *Meher* (autumn) livelihood zone demonstrated better food security. The binary logistic regression identified 12 significant determinants of food security (*P* < 0.05), including livelihood zone, education level, sex of household head, family size, off-farm income, land size, number of land plots, number of oxen, use of chemical fertilizer, household head’s health status, experience of conflict, and access to remittances.

**Conclusion:**

Household food security in the study area is significantly influenced by a complex interplay of socio-economic, agricultural, and environmental factors and poverty levels remain high in the study area. Interventions aimed at improving food security in the North-Eastern Highlands of Ethiopia should adopt a multi-faceted approach, addressing factors such as education, access to resources, agricultural productivity, and household vulnerability to shocks like conflict and illness. Collaborative efforts by government, non-governmental organizations, and local stakeholders are crucial for achieving sustainable improvements in food security.

## Popular scientific summary

- Ethiopia suffers persistent food insecurity due to drought, conflict, and resource constraints.
- A survey of 382 rural households was conducted in South Wollo Zone, North-Eastern Highlands.
- Methods: Binary logistic regression (determinants) and Foster-Greer-Thorbecke model (poverty).
- Food insecurity incidence, depth, and severity measured 55, 18, and 8%.
- Determinants included livelihood zone, education, household head’s gender, family size, land, oxen, fertilizer use, health, conflict, and remittances.
- Results emphasize multi-sector interventions in education, agriculture, health, and peacebuilding.

Food security is achieved when all individuals, at all times, have consistent physical and economic access to sufficient, safe, and nutritious food that satisfies their dietary needs and aligns with their cultural preferences, enabling them to lead active and healthy lives (1). In accordance with this definition, food security is characterized by four primary dimensions: food availability, food access, food utilization, and food stability (2). On the other hand, when people do not have adequate physical, social, or economic access to food as defined above, food insecurity will occur (2).

Food insecurity remains at severe levels where 733.4 million people are undernourished in the world in 2023. Moreover, the most recent Global Report on the Food Crisis (GRFC) predicted that high levels of acute food insecurity would cause 281.6 million people in 59 countries to need urgent food assistance in 2023 (3). Likewise, about 298.4 million undernourished people are living in Africa and 277.7 million are in Sub-Saharan Africa (SSA) (4). According to FAO et al. (5), acute food insecurity affecting 56.85 million people in eight East African countries, including Ethiopia in 2022, was mostly caused by conflict, drought, climate variability and extremes, and economic downturns and slowdowns. Furthermore, GRFC suggested that in eight East African countries including Ethiopia, the already high levels of acute food insecurity were made worse by the aftermath of the historic 2020–2023 drought, El Niño-driven floods, increased conflicts, and ongoing macroeconomic uncertainty. As per the predictions made by Beghin et al. (6), there will be a rise in the number of people facing food insecurity in SSA from around 254 million in 2013 to 373 million in 2023. Furthermore, in 2020, the region was home to above 4.4 million refugees, while almost 9.5 million people experienced internal displacement, mostly in South Sudan, Ethiopia, Somalia, and Sudan (7).

Ethiopia has the worst levels of food insecurity in the world, with high record requirements for food aid as a result of the country’s protracted drought and ongoing conflict (8). Consequently, food insecurity is a persistent and serious problem in Ethiopia, which has over 133 million people living there as of 2024, making it the second most populated country in Africa after Nigeria (9). Owing to their reliance on rain-fed agriculture and rural living, over 73% of Ethiopians are vulnerable to weather fluctuations (10, 11). Consequently, the level and scope of food insecurity in Ethiopia continue to rank among the worst globally (12). In Ethiopia, 18.7 million people were living with acute food insecurity as of 2024 (3). Moreover, the number of undernourished people in Ethiopia was 27.3 million in 2023 (4). Research suggests that the country’s food insecurity is caused by several factors, including population expansion, recurrent droughts, irregular and insufficient rainfall, a lack of advanced technologies, land degradation, and extended conflicts (13–15). Correspondingly, the conflict in Tigray and the surrounding Amhara and Afar regions has resulted in severe food insecurity, mass displacement, restricted access to services, and the collapse of the local economy (16).

Various factors determine the status of food security at the household level. For instance, Agidew and Singh (17) identified farmland shortage, poverty, recurrent drought, climate change, insufficient rainfall, and land degradation as key factors influencing food security. Moreover, according to Shumiye (18), non-participation in off-farm activities, having a large family size, low annual production or yield, small farm size, dependability attitude on food aid, poor wealth status, and insecure land tenure perception are positive and significant factors that contributed to high food insecurity.

Rigorous measurement and analysis of poverty are fundamental to evaluating the socio-economic well-being of a population (19). As Freguja and Polidoro (19) argued, poverty, hunger, inequality, and the escalating climate crisis are interconnected global challenges demanding urgent and comprehensive solutions. Diverse methodologies exist for undertaking such analysis, each offering distinctive and significant contributions to our comprehension of this complex issue. Among the various poverty measures, the Foster–Greer–Thorbecke (FGT) model, analyzes the prevalence of food insecurity (headcount index), how far below the food security threshold food-insecure households fall (food insecurity gap), and the inequality in this gap among those households (squared food insecurity gap) (20).

The South Wollo zone, where the study is located, has a serious food security issue because of its high population density, small landholdings per household, heavy reliance on highly variable summer rainfall, and diminishing soil fertility (21). According to the same author, the South Wollo zone is one of the Amhara Region’s drought-prone areas. The large number of people living in the zone are reliant on food aid due to the structural food shortfall they are going through (22).

Numerous studies have found several factors that contribute to determining the food security status of households in different parts of Ethiopia. For instance, Feyisa (23) identified factors such as education level, dependency ratio, land size, and amount of fertilizers, while Mohammed and Mohammed (24) identified livestock ownership excluding oxen, oxen ownership, cultivated land size, non-farm income, extension contact, and household head educational status. Moreover, Awoke, Eniyew (25) identified access to training, sex, family size, number of oxen, off-farm, farmland size, age, tropical livestock unit, livelihood diversification, and household on-farm income. Aragie and Genanu (26) in their parts identified monthly expenditure, age of household head, dependency ratio, non-farm income, distance from input market, family size, farmland size, number of oxen, and livestock ownership. Correspondingly, Debebe and Zekarias (27), Gazuma (28), and Arega (29) studied the incidence, depth, and severity of food insecurity in different parts of the country. However, the findings from different studies came up with significantly different results. Besides, to the best of the researcher’s knowledge, there was no study in the North-eastern highlands of Ethiopia, South Wollo to identify factors that determine food security and analyze the extent of poverty at the zone level. Hence, this study was conducted to fill the aforementioned gaps.

The general objective of this study was to identify factors that determine food security and analyze the extent of poverty at the household level in the North-Eastern Highlands of Ethiopia. The specific objectives include: 1) identifying the factors that determine household food security in the study area and 2) analyzing the incidence, depth, and severity of food insecurity in the study area.

## Conceptual framework of the study

**Fig. 1:**
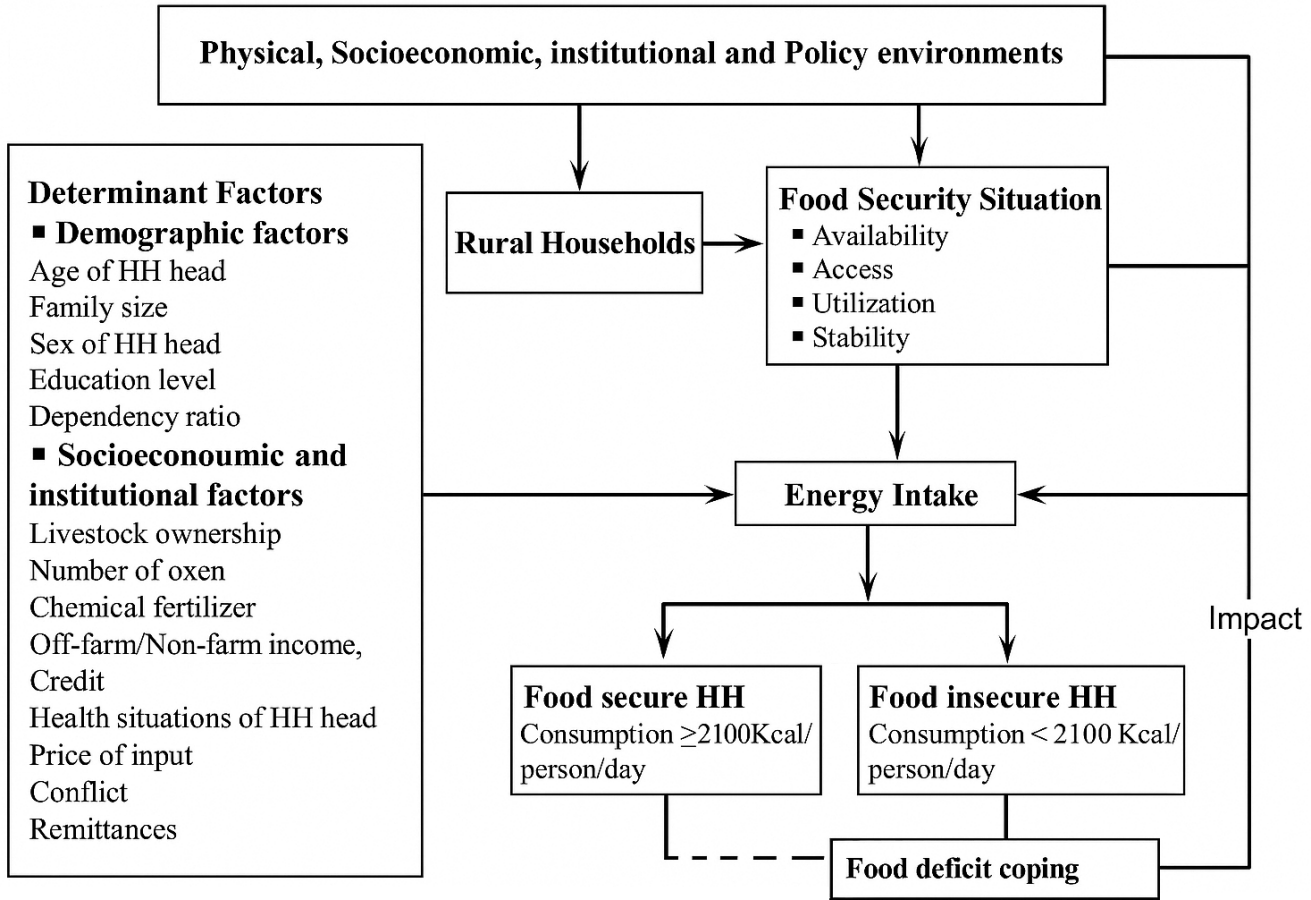
The conceptual framework of the study: Modified from Tefera and Tefera (31).

Food security is a function of several factors that include individuals and households to access nutritionally adequate and safe food. As demonstrated in Fig. 1, the physical, socioeconomic, institutional, and policy environments influence the four components of food security namely, availability, access, utilization, and stability (30). Availability refers to the physical existence of food, the production, exchange, and distribution, of food in adequate quality for people to meet basic nutritional needs (31). However, food access refers to the ability of a household to access food through production, purchase, gifts, borrowing, or aids in the required quantity, quality, and nutritional type.

Food access is linked to a household’s capacity to pay for the food required for an active and healthy life (31). The physical, social, and policy environments influence food access by affecting how well households use their resources to achieve their goals for food security (32). However, severe variations in these circumstances, like those that occur during a drought or social unrest, have the potential to substantially impair production capacity or the ability to generate income from it, endangering the affected households’ access to food. These shocks have serious ramifications for families’ productive capacity and, consequently, their long-term food security. They also harm households’ temporary access to food and frequently result in the loss of productive assets like livestock. When these conditions worsen, households may experience food insecurity and their daily calorie availability may fall below 2,100 kcal per person per day.

Likewise, good care and feeding practices, food preparation, dietary diversity, intra-household food distribution, access to clean water, sanitation, proper food processing and storage techniques, and healthcare are aspects of food utilization (5). Coming to stability, when the requirements for availability, access, and utilization are fully satisfied, the system as a whole is said to be stable, guaranteeing that households always have access to food (5). Furthermore, household energy availability is influenced by demographic characteristics, socioeconomic and institutional factors, and environmental conditions. Based on the type of influence of the determining factors, the household could be either food secure or food insecure. When households have food insecurity, they could undertake coping strategy measures to improve their food security status. As indicated by the broken line (Fig. 1), employing coping strategies could not be the practice followed only by food insecure households, but also food secure households that satisfy the daily energy needs despite also facing food shortages in some months over the year being forced to employ coping strategies. Finally, the strategies adopted by households to cope with food scarcity affect calorie availability, food security, and ultimately the physical, socioeconomic, institutional, and policy environments.

## Materials and methods

### Description of the study area

**Fig. 2:**
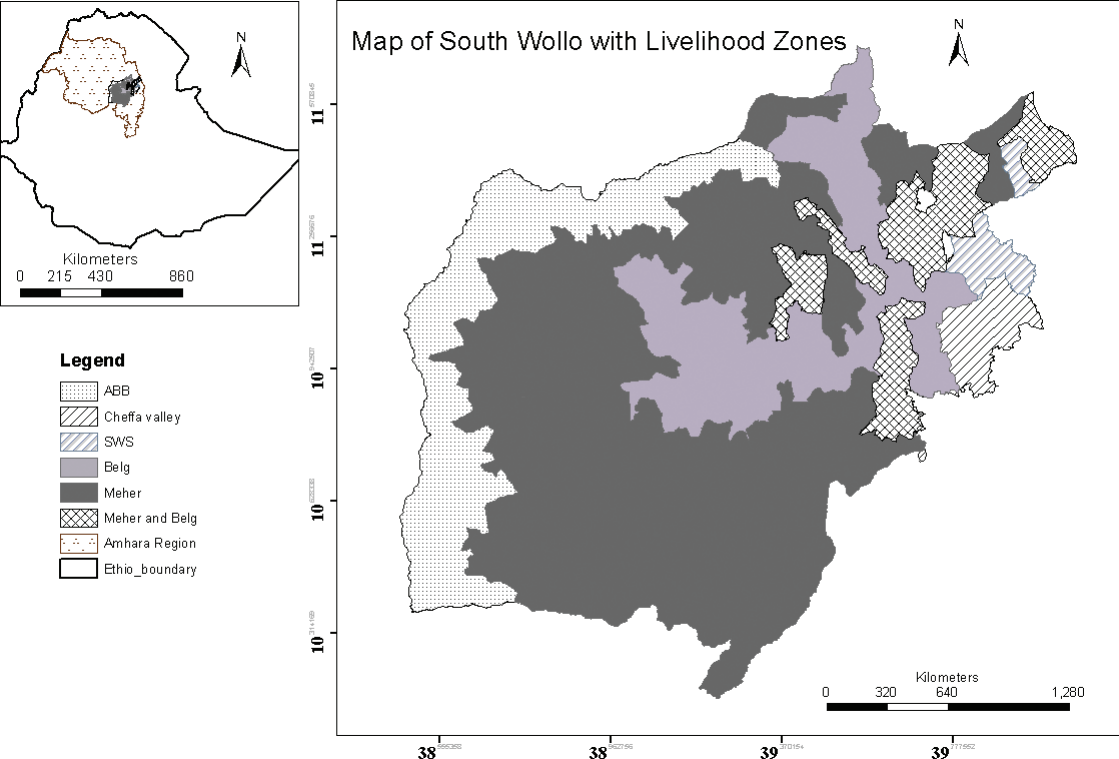
Livelihood zones map of the South Wollo zone.

This study was undertaken in the South Wollo zone of the Amhara National Regional State, Northeastern part of Ethiopia. It is specifically located between latitudes 10°10’N and 11°41’N and longitudes 38°28’ E and 40°5’E (Fig. 2). The South Wollo Zone Plan Commission (33) estimated that 3,132,062 people live in the zone in 2022, with 51% of them being men and 49% being women. The same source indicates that with a population density of 171 persons per km^2^, the zone is among the most densely populated areas in the region. Also, 86.6% of the population resided in rural areas, and the remaining 13.4% were urban residents. The zone comprised 19 rural districts and four administrative towns (Haik, Dessie, Mekaneselam, and Kombolcha) with a total size of more or less 18,157.48 km^2^ (34). Moreover, *Belg (Spring), Meher* (autumn), Abay-Beshilo Basin (ABB), *Meher-Belg*, Chefa Valley (CHV), and South Wollo and Oromia eastern lowland sorghum and cattle, are the six livelihood zones (LHZ) in South Wollo (https://fews.net/east-africa/ethiopia/livelihood-zone-map/january-2018). The zone’s primary economic activity is the agricultural sector. Since raising livestock and agriculture are the community’s main sources of income, mixed farming is the main economic activity in the area (35).

### Research methodology

#### Research design

The research philosophy used in the study was the pragmatism paradigm, which supports the use of mixed research methodologies. Within the confines of a single research, pragmatics can incorporate both positivist and interpretive viewpoints. A convergent parallel design entails the fact that the researcher simultaneously conducts the quantitative and qualitative elements in the same phase of the research process, weighs the methods equally, analyses the two components independently, and interprets the results together (36). A mixed-methods design was used to maximize the benefits of the quantitative and qualitative approach the researcher relied on for the inquiry (37–39). Pragmatism is the best research philosophical orientation since this study used a range of primary and secondary sources of information (such as survey questionnaires, key informant interviews, and focus group discussion [FGD]) and analysis procedures.

#### Sampling techniques and sample size determination

Both probability and non-probability sampling techniques were used in this investigation. The study used multi-stage sampling procedures to choose the participants who would fill out the questionnaire. The South Wollo Zone was purposely selected as the study area because it is located in the Northeastern highlands of Ethiopia, a region recognized as a food insecurity hotspot due to climate unpredictability, recurring drought, and persistent conflict. South Wollo Zone is susceptible to extreme drought conditions because of rising temperatures and falling precipitation (40) which needs a thorough investigation by policymakers. Second, this study focused on examining the factors that influence food security and the extent of poverty; yet, earlier studies conducted in the study area did not emphasize it. Third, South Wollo was selected purposively for the study mainly due to the principal investigator’s familiarity with the problems selected for the study. Most importantly, communication was not a barrier to collecting the necessary data since the author’s place of birth is the South Wollo zone specifically, in the Werebabo district.

To select the respondents to fill out the questionnaire, the study used a combination of stratified, simple-random, and systematic sampling procedures. Based on stratified sampling, the study region was divided into six livelihood zones (strata), with each group of LHZs serving as the sample strata. Next, six sample *kebele* administrations, one from each stratum, were chosen using a simple random sampling procedure. Thus, Maskeraba, Keteteye, Abahilme, Fito, Hamusit, and Yaya from the livelihood zones of ABB, SWS, CHV, *Meher-Belg, Belg (spring)*, and *Meher* (autumn), respectively, were the sample *kebeles*. A total of 8,528 rural household heads in the six sample *Kebele* administrative districts served as the sampling frame for the study. The Yemane (41) formula was used to determine sample size, as shown below:


$$n=\frac{N}{1+N{\left(\text{e}\right)}^{2}}$$ (1)

where: *n*=Sample size

*N* = total number of households = 8,528

e = Maximum margin of sample error (0.05) at the 95% confidence interval

$$\text{n}=\frac{8528}{1+8528{\left(0.05\right)}^{2}}=\frac{8528}{22.32}=382$$

Using the formula, 382 households were determined to complete the survey questionnaire. Even more than that, the proportional stratified sampling technique was used to calculate the sample size to be obtained from each *Kebele*. Lastly, based on a systematic random sampling technique, rural household heads were chosen as the sampling units based on their list from the corresponding *Kebele* administrations. As a result, 382 questionnaires were distributed to the selected respondents to be filled. Of these, 367 (96%) were returned, which were then utilized for the analysis.

The purposive sampling method was employed for qualitative sampling to choose research participants who could provide thorough and specific information. Accordingly, a total of 17 key informants (13 men and 4 women) took part in the interview, including six agricultural developmental agent officers at the *Kebele* level, six community leaders, four district-level food security officers, and one zonal-level food security officer. Six FGDs with six to seven participants per FGD at each sample *kebele* were conducted with community leaders, elderly people, leaders of women’s associations, leaders of youth associations, female-headed households, and leaders of religions.

### Data sources and data collection techniques

In this study, both primary and secondary data sources were used. Key informants, FGD participants, survey respondents, and observation were primary data sources. Reports of the Central Statistical Authority (42), reports from various administrative levels, unpublished sources, and the internet were sources of secondary data. The secondary data sources were utilized to know the number of households in each sample kebele and the socio-economic characteristics of the population. Data were gathered using the survey questionnaire, KI interview, FGD, and observation.

### Data analysis techniques

Both qualitative and quantitative data analysis techniques were used for this study. Thematic analysis, transcription, and textual analysis were used to analyze the qualitative data. For instance, thematic analysis can be applied in a variety of theoretic and epistemological contexts and is a suitable technique for understanding experiences and thoughts (43). Hence, the themes created in this study were: diminishing land size, remittance availability, occurrence of conflict, chemical fertilizer, and oxen ownership.

The quantitative data in this study were analyzed using both descriptive and inferential statistics. Inferential statistics such as T-test, one-way Analysis of Variance (ANOVA), and a binary logistic regression model were employed. T-tests and one-way ANOVAs were used to assess differences in mean energy availability across various socioeconomic and demographic categories. Descriptive statistics, including percentages and frequencies, were used to summarize the characteristics of the data.

This study employed the binary logistic regression model to investigate factors that affect household food security. Besides, the FGT model was employed in analyzing the incidence, depth, and severity of food insecurity.

### Binary logistic regression model

In this study, information on household food consumption was collected through a household survey using the table titled ‘Household Dietary Energy Supply (Kcal) Questions’. Based on these data, the total quantity of food available to each household throughout the year was first calculated in kilograms and then converted into kilocalories (kcal) using the conversion table for each food group (Appendix 1). The resulting annual household food availability in kilocalories was divided by 365 to determine the daily household energy availability, which was then adjusted by the number of adult-equivalent units to compute the per capita daily available energy. The daily net calorie availability of the household was divided by the adult equivalent (AE) to obtain the daily energy availability/kilocalorie per person per day (kcal p/p/day). Next, the kcal of each sample household was compared with the minimum subsistence requirement p/p/day. Based on the Ethiopian Health and Nutrition Research Institute (44) recommendation, households were classified as food secure (households with ≥ 2,100 kcal/p/p/day) and food insecure (households with < 2,100 kcal/p/p/day). It should be noted that the analysis by age and sex was conducted based on the household head’s characteristics rather than those of individual members. Then, the binary logistic regression model was employed to investigate factors affecting food security. Logistic regression measures the relationship between the categorical dependent variable and one or more independent variables by estimating probabilities using a logistic function, which is the cumulative logistic distribution (45). It can be used for predicting a response variable based on continuous, discrete, dichotomous, or a combination of any of these predictor variables (46, 47). Moreover, the model is important to determine the per cent of the variance in the response variable explained by the predictor variables. The model is also used to rank the relative importance of predictor variables, to assess interaction effects, and to understand the impact of covert control variables (48).

According to Costa e Silva, Lopes (49) and Schober and Vetter (50), binary logistic regression is appropriate when the dependent variable is a dummy. As the dependent variable has a dichotomous nature (food secure or insecure), a binary logistic regression is appropriate where the estimated probabilities lie between logical limits 0 and 1 (46, 47). Therefore, in this study, a binary logistic regression model was used to identify factors affecting food security. Household food security is a dichotomous dependent variable in this model.

### Dependent and independent variables

#### Dependent variables

The dependent variable of the study was a household food security status that was a dichotomous variable (food security and food insecurity) and a function of numerous explanatory variables. Hence, food security statuses as (1 = food secure households and 0 = food insecure households) are shown below.


$$\text{HFSi}=\left\{ \begin{array}{l} 0,\;{\text{Y}}_{\text{i}}<\text{R }(\text{Food insecure})\\ 1,\;{\text{Y}}_{\text{i}}\geq \text{R }(\text{food secure}) \end{array}\right.$$

HFSi = household food security status of the i^th^ household, i = 1, 2, 3, 4, ……………., 367

Yi = daily per capita calorie availability (supply)

*R* = the minimum recommended national standard rate of calories per person per day (2,100 kcal)

**Independent variables:** Drawing from the existing literature, we selected a set of independent variables, measured on either a categorical or continuous scale, to examine their influence on household food security (Table 1).

**Table 1:** Summary of predictor variables used in the logistic regression model

Variables	Type	Description	Excepted sign
Livelihood zone	Categorical	1 = Abay-Beshilo Basin (ABB), 2 = South Wollo and Oromia eastern lowland sorghum and cattle (SWS), 3 = Chefa Valley (CHV), 4 = *Meher-Belg*, 5 = *Belg(spring), 6 = Meher(autumn)*	+_
Sex of the household head	Dummy variable	1 = male, 2 = Female	+
Age of the household head	Continuous variable	Age of the household head	-
Education of the household head	Categorical	1. Unable to read & write, 2. Read and write, 3. Grade 1–8, 4. Grade 9–12, 5. Diploma & Above	+
Family size	Number	The number of people in the household	-
Age dependency ratio	Continuous	The ratio of persons under 15 and above 65 years old to those (15–65 years old)	-
Off-farm income	Continuous	The amount of income a household received in the year from off-farm activities (ET Birr)	+
Farm land size	Continuous	Land size in hectares	+
Number of farm plot	Number	The number of plots a household has	+
Soil fertility	Dummy	(1 = Good, 2 = Bad)	+
Number of oxen	Number	The number of oxen	+
Livestock ownership	Continuous	TLU (Tropical Livestock Unit)	+
Chemical fertilizer	Dummy	(1 = yes, 2 = no)	+
Price of the input	Continuous	Cost per year for input in ET. Birr *	-
Crop disease	Dummy	(1 = yes, 2 = no)	-
Credit amount	Continuous	The amount of credit available for the household in the year (ET Birr)	+
The health situation of the household head	Dummy	(1 = Good, 2 = Bad)	+
Conflict	Dummy	(1 = yes, 2 = no)	-
Remittances	Dummy	(1 = yes, 2 = no)	+

* 1$=56.58 ET Birr

### Assumptions

Assumptions that must be met for logistic regression analysis in this study include the independence of observations in the dataset and the absence of multicollinearity among the explanatory variables. The presence of multicollinearity was checked based on the commonly used cut-off points (tolerance value of less than 0.1, or a Variance Inflation Factor (VIF) value of above 10). Moreover, the lack of strongly influential outliers and the existence of a linear relationship between each explanatory variable and log-odds of the response variable were some of the assumptions.

One binary-dependent variable with two categories (food security status) and several independent variables (predictors) were proposed for analysis in this study. Therefore, the binary logistic regression model that was applied to screen out the most significant variables of the study is described as (Equation 7.2):


$$\begin{array}{l} \ln Y=\ln\left[\frac{Y}{1-Y}\right]=\beta_{0}+\beta_{1}X_{1}+\beta_{2}X_{2}\\ \;+\beta_{3}X_{3}+\ldots +\beta_{n}X_{n}+ui \end{array}$$ (2)

where: ln = natural logarithm, Y = probability of being food secure, 1–Y = probability of being food insecure, βn<comp: please make ‘n’ as subscript> = coefficients of explanatory variables, Xn<comp: please make ‘n’ as subscript> = predictor variables, and *ui* < comp: please make ‘i’ as as subscript > = error term.

### Assessment of the goodness-of-fit of the model

Checking the goodness-of-fit is imperative for the binary logistic regression model. The Pearson x^2^ statistics and Hosmer–Lemeshow (HL) tests are the two well-known statistics used to test that the observed data came from a population in which the fitted logistic regression model is true (51). Pearson x^2^ will be calculated to evaluate the discrepancy between predicted and observed counts within the groups (52). According to Nattino, Pennell (53) and Das (54), the use of the x^2^ statistics of the logistic regression model is better and easier to visualize and compute by handling the data as a binary response through different values of the contingency table.

Moreover, the HL goodness-of-fit test is equivalent to testing the hypothesis that the observed number of events in each of the groups is equal to the expected number of events in the fitted model (53). Besides, a test of the null hypothesis about the goodness-of-fit of the model that fits the data against the alternative hypothesis was tested by the HL test (51). Besides, another way of evaluating the goodness of fit of a given logistic regression model is a classification table. The classification table is a 2 × 2 contingency table of the observed and predicted results. The model was used to classify each record using the computed probabilities ranging between 0 and 1 with a 0.50 minimum probability or cut value (55). Furthermore, the pseudo-R squared was used to test the goodness of fit in the model. It is the proportion of the variance of the latent variable that is explained by the culvert. Pseudo-R squared values were used when the outcome variable is nominal or ordinal such that the coefficient of determination R squared cannot be applied as a measure for the goodness of fit (51).

### The Foster–Greer–Thorbecke poverty measures

The FGT model examines the household headcount index (incidence of food insecurity), food insecurity gap (depth of food insecurity), and the square of the food insecurity gap (severity of food insecurity) among the food insecure households (20). According to Debebe and Zekarias (27), the FGT index measures the mean of household food insecurity gaps raised to the aversion parameter *a*, which is the severity of food insecurity. The FGT formula was used to measure how income was distributed around a poverty line or threshold that describes how many poor are falling far below the poverty line and how many are hovering near it (56). The mathematical formula of the FGT model is specified as follows in equation 6.3.


$$\begin{array}{l} F(\alpha )=\frac{1}{ni}\sum_{i=1}^{q}{\left[\frac{m-yi}{m}\right]}^{\alpha }\\ \;=\frac{1}{n}\left[\frac{{\left(m-y1\right)}^{a}}{m}+\frac{{\left(m-y2\right)}^{a}}{m}+\ldots \frac{{\left(m-yn\right)}^{a}}{m}\right] \end{array}$$ (3)

where: n = the number of sample households, *q* = the number of food insecure households, m = the cut-off between food security and food insecurity (expressed here in terms of caloric requirement), yi<comp: please make ‘i’ as subscript> = the food calorie availability per adult of the i^th^ household, and a = the weight attached to the severity of food insecurity.

Therefore, according to Pignataro and Costa (20), if m < yi<comp: please make ‘i’ as subscript>, the household is food secure, and if m > yi<comp: please make ‘i’ as subscript>, the household is food insecure, and if α = 0 then F(α) = q/n. In the equation, (m–yi<comp: please make ‘i’ as subscript>) = 0 if yi<comp: please make ‘i’ as subscript> > m. That means it is possible to consider (m–yi<comp: please make ‘i’ as subscript>) = 0 when the household has enough food energy and it is food secure. In this study, three steps were followed to measure the incidence, depth, and severity of food insecurity: first, food supply at the household level was determined by compiling a food balance sheet for each household; second, the available grain was converted to total calories available to each household using a kilocalorie conversion Table (Appendix 1). By using the calculated food supply at the household level, calories available per AE per day for each household were calculated. Then, 2,100 kcal/p/d was employed as a threshold between food-secure and food-insecure households. Third, the FGT index was used to estimate the food insecurity incidence, gap, and severity among the surveyed rural households.

## Results and discussion

### Demographic and socio-economic characteristics of respondents

The food security situation of respondents based on the kcal indicator per different demographic and socioeconomic characteristics was summarized in Table 2. The majority (68%) of the respondents in the study were males and 32% were females. The mean energy availability of males was significantly larger (1,991 kcal) than that of females (1,692) and the difference was significant at *P* < 0.01 (Table 2). Hence, female-headed households are more food insecure than male-headed households. The result shows the majority (83.5%) of the households surveyed belonged to the productive age group of 35–64. Contrasting age categories with food security status in kcal consumption per person per day, the mean for the age group ≥ years was the greatest (2,080 kcal) followed by the 35–49 age group and the 50–64 age group with mean kcal of 1,946 and 1,807, respectively (Table 2).

**Table 2:** Demographic and socio-economic characteristics of respondents and per capita kcal consumptions (*n* = 367)

Variables	Category	Percent of total	Per capita kcal consumption per day				Test statistic
			Minimum	Maximum	Mean	Range	*P*-value One-way (ANOVA)
Age group	20–34	4.5	509	3,105	1,651	2,597	0.033**
	35–49	47.5	299	3,690	1,946	3,391	
	50–64	36.0	460	3,470	1,807	3,010	
	≥65	12.0	769	3,691	2,080	1,311	
Family size	1–2	4.6	1,030	3,691	2,414	2,661	0.001***
	3–4	22.6	416	3,652	2,013	3,236	
	5–6	51.5	299	3,650	1,877	3,351	
	7–8	18.8	409	2,756	1,711	2,347	
	≥9	2.5	837	2,305	1,835	1,468	
Sex	Male	68	299	3,690	1,991	3,391	0.000*** (T-Test)
	Female	32	460	3,691	1,692	3,231	
Marital Status	Married	74.9	299	3,690	1,952	3,391	0.008***
	Single	6.0	777	2,887	1,653	2,110	
	Divorced	9.5	694	3,470	1,933	2,776	
	Widowed	9.5	460	3,691	1,897	3,231	
Education level	Unable to read and write	42.2	409	3,691	1,843	3,281	0.000***
	Read and Write	25.6	299	3,470	1,796	3,171	
	Grade 1–8	20.7	460	3,647	1,902	3,187	
	Grade 9–12	8.4	942	3,690	2,263	2,748	
	Diploma & above	3.0	1,796	3,652	2,508	1,856	
LHZ	ABB	10	832	2,560	1,822	1,727	0.000***
	*Belg* (spring)	19	460	2,433	1,313	1,973	
	CHV	17	299	3,691	1,842	3,392	
	*Meher* (autumn)	17	409	3,690	2,388	3,281	
	*Meher-Belg*	16	817	2,664	1,973	1,847	
	SWS	21	834	3,412	2,034	2,578	
Food security status (Kcal)	Food secure	45.5	2,102	3,691	2,475	3,392	0.000*** (T-Test)
	Food insecure	54.5	299	2,094	1,419	1,795	

***, **, * significant at *P* < 0.01, *P* < 0.05, and *P* < 0.1 respectively.

The ANOVA test result reveals that there was a significant difference in the mean kcal conception between age groups at *P* < 0.05. With increasing age groups, mean kcal was slightly decreased. The result agrees with some studies in developing countries (57–59). However, the test result disagrees with the studies conducted in Refs. (25, 60, 61). Moreover, 74% of the households had a family size of three to six, 75% of the households were married, and nearly 68% of the respondents were not enrolled in any formal education (Table 2).

Mean daily energy availability, measured in kcal/person/day, decreased as household size increased. Households with 1–2 members had the highest availability (2,414 kcal/person/day), indicating a greater level of food security compared to households with 3–4 members (2013 kcal/person/day) and 5–6 members (1,877 kcal/person/day). The mean energy availability difference between family size groups was found to be significant at *P* < 0.01 (Table 2). This event suggests that food security decreases with increasing family size which agrees with the findings in Ref. (62).

Likewise, married households had the largest mean kcal consumption having 1,952 kcal/p/d followed by divorced and widowed with mean kcal/p/d 1,933 and 1,897; whereas, singles had the least mean kcal consumption. Based on the ANOVA test result, there is a significant difference at *P* < 0.01 in mean kcal consumption between the marital status groups (Table 2). Rural households used to take on the responsibility of farming activities after getting married. According to Doukoro, Abbey (63), and Mota, Lachore (64), much of the rural agricultural livelihood activities are practiced by married people. The comparison of education level with kcal consumption showed that the highest records were with households attaining the diploma and above followed by grades 8–12 and 1–8 having mean kcal 2,508, 2,263, and 1,902 kcal/p/d respectively (Table 2). Moreover, the difference in mean kcal between education level categories was significant at *P* < 0.01. Households unable to read and write and read-and-write education levels have the lowest kcal consumption. From this result, it is evident that education level has a high relation with food security, and hence households without formal education are more vulnerable to food insecurity than others.

Furthermore, households living in the *Meher (autumn)* LHZ were the most food secure with 2,388 mean kcal/p/d followed by SWS with 2,034 kcal (Table 2). On the contrary, *Belg* (spring) livelihood zone was the most food insecure with a mean kcal consumption of 1,313. Correspondingly, the mean kcal difference among livelihood zones was significant at *P* < 0.01. Finally, by taking the cut-off point (2,100 kcal/p/d) to examine households’ food security status; the majority (55.5%) of households were food insecure and lay below the benchmark. The T-test result reveals that there is a significant difference in mean kcal between food-secure and food-insecure households at *P* < 0.01 (Table 2).

### Factors affecting household food security

The binary logistic regression model was used to establish the relationships between food security and a set of predictor variables. It was selected as it can be used with continuous, discrete, and dichotomous variables mixed (46, 47). Assumptions such as multicollinearity, independence of observation, and outliers were checked and there were no significant violations of the stated suppositions. Likewise, the model was checked for goodness of fit by using different methods. In all standards, the model was found appropriate and well-fitted the data employed (Table 3). The likelihood ratio (LR) test of model coefficients has a Chi-square value of 439.77 on 27 degrees of freedom, which is strongly significant at *P* < 0.001 indicating that the predictor variables have a higher joint effect in predicting the status of household food security. The dependent variable is explained by all the independent variables. The HL test indicates a poor model fit if the significance value *P* < 0.05. But in this model, the HL Chi-square statistic test was 0.8 with *P* > Chi-square = 0.9989. Hence, as the significance value is greater than 0.05, the model is adequately fitted indicating that there is no difference between the observed and predicted model. The pseudo *R* squared statistic was 0.8695 showing that almost 87% of the likelihood of a household being food secure was strongly explained by the predictors. Moreover, the predictive efficiency of the model showed that out of the 367 samples included in the model, 354 (96.46%) were correctly predicted which is much higher than the cut-off point of 50%. The sensitivity (correctly predicted food security) and specificity (correctly predicted food insecurity) were found to be 95.5 and 97.6%, respectively. Therefore, the discussion and interpretation of the explanatory variables in the model are presented hereunder.

**Table 3:** Factors affecting household food security (using a binary logistic regression model)

Variables	Odds ratio	Std. Err.	z	*P* > z	[95% Conf.	Interval]
**LHZ (ABB = RC)**						
SWS	234.6862	556.544	2.30	0.021**	2.248614	24494.02
CHV	1399.859	4468.347	2.27	0.023**	2.685662	729654.7
Meher-Belg	43.36031	89.85316	1.82	0.069*	0.746784	2517.618
Belg (spring)	0.2109313	0.4371022	−0.75	0.453	0.0036328	12.24737
Meher (Autumn)	160.6545	356.5622	2.29	0.022**	2.07351	12447.43
**Sexhead**						
Female headed	0.0982034	0.1029712	−2.21	0.027**	0.0125776	.7667511
Agehead	0.9901431	0.0487045	−0.20	0.840	0.8991413	1.090355
Dependency Ratio	1.012449	0.0092283	1.36	0.175	0.9945227	1.030699
**Education level (Unable to read and write = RC)**						
Read and Write	0.2688836	0.2952184	−1.20	0.232	0.0312604	2.312777
Grade 1–8	0.3830207	0.5085857	−0.72	0.470	0.0283774	5.169778
Grade 9–12	288.7822	541.7906	3.02	0.003***	7.304869	11416.38
Diploma and above	11.03813	79.04379	0.34	0.737	8.86e−06	1.38e+07
Family size	0.3718857	0.1479031	−2.49	0.013**	0.17056	0.8108526
**Crop disease**						
No	2.902928	2.996647	1.03	0.302	0.3838437	21.95423
Off-farm income	0.19092	0.1909436	−1.66	0.098*	0.0268871	1.355683
Land-size	49.11795	88.77071	2.15	0.031**	1.421891	1696.735
Number of plot	2.387017	1.148524	1.81	0.071*	0.9295997	6.129359
**Soil Fertility**						
Bad	0.233806	0.2752264	−1.23	0.217	0.0232733	2.348842
Number of Oxen	180.0971	256.0117	3.65	0.000***	11.10468	2920.836
Livestock ownership (TLU)	1.68855	0.7408541	1.19	0.232	0.714575	3.990065
**Chemical fertilizer**						
No	0.0181738	0.0297909	−2.44	0.014**	0.0007314	0.4515974
Price of input	0.9995953	0.0006013	−0.67	0.501	0.9984174	1.000775
**Credit amount**						
No	2.055396	1.654943	0.89	0.371	0.4241638	9.959955
**Health situation of the head**						
Bad	0.0037613	0.004866	−4.32	0.000***	0.0002979	0.0474831
**Conflict**						
No	28.14081	44.22087	2.12	0.034**	1.29344	612.2475
**Remittances Availability**						
No	0.0339744	0.0342275	−3.36	0.001***	0.0047164	0.2447342
cons	0.0374521	0.1321204	−0.93	0.352	0.0000372	37.69442
Number of observations						367
LR Chi-square (26)						439.77
Prob > Chi-square						0.00000
Log-likelihood						−33.0127
Pseudo R2						0.8695
Sensitivity						95.50
Specificity						97.60
Correctly classified						96.46

***, **, * significant at 1, 5, and 10% level respectively.

Variables assumed to influence household food security in different contexts were tested in the regression model, and out of 19 variables 12 of them were found to be statistically significant at 10, 5, and 1% probability levels (Table 3). These include LHZ, sex, education level (10–13), family size, off-farm income, land size, number of plots, soil fertility, number of oxen, and the health situation of the household head. Correspondingly, the use of chemical fertilizers, conflict, and remittance availability were significant predictors of food security. Hence, based on Table 3, variables that had a significant influence on food security are discussed further in the text.

### Sex of household head

The result revealed that female-headed households are less likely to be food secure with the odds ratio of 0.09820 as compared to male-headed households and it was significant at *P* < 0.05 (Table 3). Females are the primary breadwinners in their households, who confront several difficulties, including a lack of time, a lack of devices that are appropriate for their physical condition, and restricted flexibility to travel outside the neighborhood to engage in various activities (65). In addition to the productive role, women are also responsible for sustaining the reproductive. The result was in agreement with Awoke, Eniyew (25) and Faustine (66). However, in contrast to the findings of Sekhampu (67), who found that because female-headed households made better use of their resources, they were more likely to be food secure than male-headed households.

### Family size

Consistent with the hypothesis, the effect of family size on food security was found to be negative and statistically significant at *P* < 0.01. The odds ratios in favor of being food secure decreases by a factor of 0.37189 with an increase in the household size by one member and other things are kept constant (Table 3). This finding agrees with prior studies such as those in Refs. (57), Akukwe (68) and Yehuala, Melak (62), who came up with similar findings. Moreover, this finding conforms to the theory of Malthus (69) with the argument that a large population lowers agricultural productivity and food security but contradicts the theory of Ref. (70) that argued that a large family size would increase agricultural productivity through intensification.

#### Education level (unable to read and write = RC)

As the regression result, households attaining grades 9–12 are more likely to be food secure with the odds ratio of 288.7822 and significant at *P* < 0.01 (Table 3). Attaining a diploma and above education level also had a positive association with food security, but not significant. However, households with no formal education were less likely to be food secure. The possible explanation is that household heads that attained secondary education had better working efficiency, competency, diversified income, and adoption of technologies to enhance production and food security than those who were unable to read and write and attained primary education. The overall result is in line with the previous studies (23, 71, 72). However, the result in the case of primary education (1–5, 7–9), contradicts Asenso-Okyere, Mekonnen (73), who found that the odds of household heads having primary education are about 2.2 times more likely to be food secure than those with no formal education.

#### Number of oxen owned

The coefficient of the number of oxen owned by the household was positive and statistically significant at *P* < 0.001 (Table 3). In the regression analysis, the odds ratio for food security increased by a substantial 180.1 for each additional ox owned, controlling for the influence of other variables. Therefore, a farmer having a pair of oxen is better food secure than a farmer without a pair of oxen. The finding of the study is consistent with prior findings of Awoke, Eniyew (25, 74, 75). Data collected through FGD also confirmed that farmers who have more oxen have the chance to plow their land plots on time and hence able to get better harvests than those who do not have oxen. Thus, they have better food security. Related to oxen ownership, a key informant from Abahilme *Kebele* (CHV LHZ) expressed his opinion:

……. Two years ago, desperation drove me to sell an ox to feed my starving family. Now, replacing that ox is beyond my reach; the price is 80,000–100,000 ET Birr. Because I lack an ox, I am unable to plough and sow my fields at the right time, perpetuating a cycle of poverty. In contrast, those who possess oxen are able to cultivate their land effectively, yielding bountiful harvests and escaping the hardships I face.

The interview result supported the regression result which revealed that lack of oxen is becoming a major challenge for the poor households in their attempt to improve their food security through ploughing and harvesting their farm. Moreover, the alarming rise in oxen price left farmers unable to replace their sold oxen. Therefore, oxen ownership in the study area’s households has significant impact on improving food security.

#### Off-farm income

Inconsistent with the prior hypothesis, the amount of income received by households from off-farm activities was negatively associated with food security. The probability of households being food secure decreased with an odds ratio of 0.1909 and significant at the *P* < 0.1 level (Table 3). The result was in line with studies by Endiris, Brehanie (76) and Sani, Mansor (77) that found households’ participation in off-farm and non-farm activities to improve the food security status at the household level was an important predictor of food security.

#### Chemical fertilizer utilization

The result revealed that households who did not use fertilizers are less likely to be food secure with the odds ratio of 0.0182 as compared to user households and it was significant at *P* < 0.05 (Table 3). The result is consistent with the hypothesis and prior studies such as those by Fikire and Bekele (60), Eneyew and Bekele (61), and Beyene and Muche (75) who came up with similar findings. However, it is against the empirical findings of Negash and Alemu (74), who in their study in rural areas of Tigray, found that fertilizer utilization is positively associated with food insecurity. However, in the study area, lack of access and the high price of chemical fertilizer harmed the agricultural yield of farm households. An elderly key informant from Fito *kebele (Meher-Belg)* stated the situation as follows:

… I remember a time when the soil was rich and fertile, and I could rely on a good harvest using only animal manure. Today, the soil’s fertility has degraded significantly due to erosion and constant ploughing. Compounding this issue, I no longer have enough animals to produce the manure needed to fertilize my fields adequately. As a result, many farmers, including myself, depend on chemical fertilizers. Unfortunately, in the past two years, fertilizer shortages have become severe, forcing us to purchase them at inflated prices on the black market. This limited access and the exorbitant prices have drastically reduced our agricultural output.

Based on the interview and the regression result, utilization of chemical fertilizers has a significant impact on increasing soil fertility and productivity and hence improves food security of households. However, the interview result revealed that availability of fertilizers in the market and the alarming increasing of its price is the great challenge facing farmers.

#### Health situations of household heads

Based on the regression result the health situation of household heads had a positive association with food security as the prior expectation and was significant at *P* < 0.01. The probability of households with bad health situations being food secure decreased by an odds ratio of 0.0038 compared with households with good health (Table 3). The health situation of household heads as well as members is the most important factor as achieving food security is difficult without proper health. The result agrees with the finding of Mohammed and Mohammed (24), who found that health status was associated with food security status.

#### Remittances availability

In line with the prior hypothesis, the regression result for remittances availability suggested that remittances have a significant positive association with food security. The odds ratio in favor of being food secure for non-beneficiaries of remittance decreased by a factor of 0.0340 and is significant at *P* < 0.01 (Table 3). That means a household that receives a remittance is more food secure than those who do not receive it. The result is consistent with the findings by Obi, Bartolini (78), and Szabo, Ahmed (79). Likewise, the data gathered through key informant interviews conformed to similar results and supported the finding. An explanation given by a *Kebele* development agent from Maskeraba *Kebele* (ABB LHZ) on the observed benefits of remittances was as follows.

… Many residents of our Kebele, particularly young people, have sought opportunities in Arab countries, both legally and illegally. While some migrants face hardships during their journeys and in the workplace, those who find success are able to significantly improve their families’ lives through remittances. This money allows them to support their siblings’ education, provide for their families’ basic needs, and even build homes. Furthermore, their investments contribute to local development. Return migrants and families use the money to start businesses, like shops, and purchase vehicles like cars and Bajajs (auto rickshaws), creating employment opportunities for themselves and their relatives. Consequently, families receiving remittances generally experience improved living standards and greater food security than others in the Kebele.

As suggested by the key informant, the remittance received from abroad particularly from Arab countries played a significant role in improving the food security situation and accumulating additional assets. Moreover, the local people are getting job opportunity using the remittances they received from their families and relatives abroad.

#### Conflict

As shown in Table 3, households who did not encounter conflict were more likely to be food secure with an odds ratio of 28.1408 compared with households that encountered conflict in the last 3 years. This is significant at *P* < 0.05 and consistent with the hypothesis. The result shows that conflict affects the supply of food by interfering with agricultural output, restricting access to land, raising food costs, and decreasing the variety of a household’s diet. The finding is consistent with studies by Lin, Kafri (80), Muriuki, Hudson (81), and Ujunwa, Okoyeuzu (82) that found food security was significantly impacted negatively by armed conflict. As to the impacts of conflict on food security, a key informant from Hamusit *Kebele* (*Belg* LHZ) expressed his opinion:

The armed conflict that raged across Northern Ethiopia in 2021/22 brought untold devastation to my family and community. Our area became a battlefield, and my house was destroyed by artillery fire. I watched helplessly as my oxen and cow were struck down. The flames consumed all our possessions, leaving us with nothing but the clothes on our backs. We fled into exile, my wife, my five children, and I. My mother, old and frail, was not so fortunate; she was shot and killed. Returning home, we found only ruins. We have no house, no animals to work the fields. And the conflict continues, making it impossible to rebuild our lives and feed our starving family.

As can be shown from the interview, the continued armed conflict that occurred in 2021/22 in northern Ethiopia (Tigray, Amhara and Afar Regions) had a devastating impact on households, stripping them of their assets, forcing them into exile, and significantly worsening their food security in the last consecutive years.

#### Livelihood zone (ABB = RC)

The results of the study showed that livelihood zones in the study area were positively associated with household food security except for the *Belg* livelihood zone. All living areas, except *Belg*, have statistical significance at the 5–10% level. The probability of a household being food secure in the *Belg* livelihood zone decreased by 0.2109, but the change was not significant (Table 3). This result is consistent with the descriptive statistics showing that households living in the *Belg* LHZ were the least food secure and consumed the least calories on average compared with households in other LHZs.

#### Number of plots

The regression result reveals that households with a large number of plots are more likely to be food secure with an odds ratio of 2.3871 for a unit increase in the number of plots with other variables controlled for (Table 3). This fact is also significant at the 10% level and consistent with the prior hypothesis.

#### Farmland size

The logistic regression results revealed that for a unit increase of farm land size, food security increases by the odds of 49.1180 when other variables are controlled for. This is also significant at *P* < 0.05. This result is consistent with previous studies by Fikire and Bekele (60) and Ferede (57). In the study area, the diminishing land size as a result of the high population is becoming a critical problem. This hinders farmers from diversifying crops and breeding livestock. An elderly key informant from Yaya *Kebele* (*Meher/* autumn LHZ) shared his opinion:

I grew up on a large family farm, where we cultivated many crops and raised numerous animals on extensive grazing lands. Now, that land has been divided among me and my six brothers. My share is so small that I can’t grow enough food to feed my family, and my children can’t even find space to build a house. This diminished land size is the root cause of our food shortages.

The decreasing trends of farm land size owing to increasing population number is reported as a great challenge for farmers to increase production and improve food security. This was revealed by the regression result and supported by the interview result as shown earlier in the text.

### Incidence, depth, and severity of Food Insecurity (FGT) poverty measures

As shown in Table 4, the incidence of food insecurity based on the energy availability approach was about 55%, demonstrating that only 45% of the households had access to the minimum required calorie recommended for subsistence. The depth of food insecurity in this study was 0.18 (Table 4). This implies that the sample households have to be provided with 18% of the daily minimum required calories to avoid the food insecurity problem. The size of the calorie deficiency gap for the sampled households was found to be 378 kcal/p/d. This suggests that, on average 378 kcal/p/d of additional food energy would be required to withdraw the households from the vicious cycle of food insecurity. The result was much lower than other related studies such as those by Jabo, Ismail (83), Arega (29), and Shimeles, Janekarnkij (84), which was 735, 819, and 483 kcal per AE per day, respectively. This was higher than the findings by Debebe and Zekarias (27), Mitiku, Fufa (85), and Olive, Aloysius (86), which were 229, 260, and 116 kcal/p/d, respectively.

**Table 4:** Incidence, depth, and severity of food insecurity in the study area (*n* = 367)

LH zone	Incidence %	Depth %	Severity %
*Belg(spring)*	79.0	39.0	21.0
CHV	60.0	20.0	9.0
*Meher-Belg*	52.0	11.0	3.4
*Meher(autumn)*	25.0	7.3	3.0
ABB	64.9	17.0	6.3
SWS	51.0	12.0	4.0
Average	55.0	18.0	8.0
Male headed	45.4	15	7.0
Female-headed	75	24	11

Moreover, the severity of food insecurity (calorie deficiency) was 0.08 (Table 4). This indicates the square of the food insecurity gap (severity of food insecurity) among the food insecure households was 8%. The severity level was also much lower than those reported by Gazuma (28), Shimeles, Janekarnkij (84), and Gebre (72) which were 25.6, 11, and 9.4% respectively. However, this was higher than some findings like Debebe and Zekarias (27), Zerihun and Getachew (87), Olive, Aloysius (86), and Mitiku, Fufa (85), which were 4.3, 1.8, 5.5, and 7.35% respectively. Having these findings in mind, the extent of food insecurity in the study area is severe and horrifying and needs policy issues at least to reduce this difficult situation.

Likewise, the incidence, depth, and severity of food insecurity were examined across LHZ and male and female-headed households. Thus, as shown in Table 4, the incidence of food insecurity was higher in the *Belg (spring)* LHZ (79%) followed by the ABB (64.9%), but lower in the *Meher (autumn)* LHZ (25%). Correspondingly, the depth of food insecurity was highest in the *Belg* LHZ (39%) and lower in the *Meher* LHZ (7.3%). The severity of food insecurity was also the highest in the *Belg* LHZ (21%) followed by the CHV LHZ (9%).

As the summarized pieces of evidence from FGD and KIs, in the *Belg* LHZ, the major factors for the severity of food insecurity were a minimizing trend of rainfall, lack of spring rain for the last 20 years, very limited or lack of off-farm and non-farm activities, the pests, the problem of infrastructure due to mountainous and rugged topography. Besides, high erosion, land degradation, and lack of agricultural input and credit were factors contributing to food insecurity. Besides, the researcher also observed the problems that aggravated the severity of food insecurity such as the ruggedness of the land topography, inaccessibility of the area for transportation, deforestation, and land degradation in the *Belg* LHZ during the field survey.

Furthermore, the incidence of food insecurity was higher for female-headed households (75%) than for male-headed households (45.4%). Likewise, both the depth and severity of food insecurity were higher for female-headed households which are 25 and 15% depth and 11 and 7% severity for females and males respectively. The result agrees with the findings by Arega (29), Modirwa and Oladele (88), and Mallick and Rafi (89) who found that the incidence of food insecurity was higher for female-headed households than for male-headed households. The mean, maximum, and minimum, of the energy availability of the sample households in this study were 1898, 3690.5, and 299 kcal respectively. The mean was 9.6% below the national minimum recommended daily allowance of energy availability (2,100 kcal).

## Conclusions

This study focuses on identifying factors that determine food security and measuring the incidence, depth, and severity of food insecurity using binary logistic regression and FGT models. Analysis of demographic and socioeconomic factors revealed that female-headed households, unmarried individuals, large families, and those with limited literacy were more vulnerable to food insecurity. Logistic regression identified 12 significant predictors of food security, including livelihood zone, education level, sex of household head, and access to off-farm income, land size, livestock ownership, fertilizer use, health, remittances, family size, and conflict. Positive associations with food security were observed for livelihood zone, education, male gender, off-farm income, land size, soil fertility, livestock, fertilizer, and household head health, while family size and conflict had negative associations.

The analysis indicated a severe food insecurity situation in the study area, with an incidence, depth, and severity of 55, 18, and 8%, respectively, particularly affecting households in the *Belg* livelihood zone, ABB livelihood, and female-headed households. The study emphasizes the need for multi-faceted interventions focusing on family planning, education and vocational training, and agricultural input access. Recommendations include the fact that regulation related to land markets should be improved by the regional and national government via improving land administration regulations, healthcare facilities and services should be improved by the districts health office and regional health bureau, policies on migration and remittances should be given due consideration by the national government and ministry of foreign affairs, and conflict resolution through government, local, and community collaboration to sustainably enhance food security and reduce poverty in the region.

## Supplementary Material



## Appendix 1

### Kilocalorie Conversion Table

**Table A1:** Representative caloric values of different foods (kcals per kg of food)

**Cereals**	**Kcal**
barley	3390
bulgur wheat	3500
maize, white, whole	3630
maize meal, refined 60-80%	3600
millet flour	3650
millet, whole grain	3630
oats	3880
pasta	3420
rice, parboiled, lightly milled	3540
rolled oats	3630
sorghum	3550
sorghum flour	3530
teff, whole grains	3450
wheat, whole	3440
wheat flour	3480
**Roots and tubers**	
cassava flour	3420
cassava, fresh	1530
plantain, ripe, raw	1280
potato raw	750
sweet potato, pale raw	1140
taro	1130
yam, flour	3170
yam, fresh	1040
**Grain legumes**	**Kcal**
beans, dried	3390
cowpeas, dried	3400
lentil, dried	3390
soya bean, dried	3820
**Vegetables**	
leaves, dark green	480
leaves, medium green	280
leaves, light green	230
maize, immature on cob	1230
onions	480
pumpkin	360
tomato	200
**Sugars**	**Kcal**
sugar	4000
soft drinks, commercial	450
**Meat, poultry, eggs, fish**	
beef, mod fat	2350
beef, lean	2020
goat meat	1450
pork, lean	3710
poultry	1390
eggs	1580
fish, dried	3090
fish, fresh	950
**Fruit**	
apple	610
avocado	1650
banana	1160
citrus	530
mango	630
papaya	390
**Milk**	
milk, cow whole	640
milk, goat whole	710
milk, sheep whole	1080
DSM vit A-enriched	3550
**Oils and fats**	**Kcal**
butter	7450
ghee	8280
margarine	7650
vegetable oil	9000
**Other**	
beer, local	350
honey	2860
**Nuts and seeds**	
groundnut, dried	5790
groundnuts, fresh	3320
pumpkin seeds (no coat)	6100
