# Supplementary material for: Factors affecting food security and poverty analysis among rural households in the North-Eastern Highlands of Ethiopia
Source: Food Nutr Res. 2025 Nov 10;69:10.29219/fnr.v69.12014. doi: 10.29219/fnr.v69.12014 (PMC13366815; doi:10.29219/fnr.v69.12014)
Supplement: Supplementary file 1 [file FNR-69-12014-s1.docx]

**Household dietary energy supply (kcal) related Questions**

What were the different sources of food for your family during 2022/23 cropping year?

| **Food item** | **Total amount using kilo gram or quintals** | | | | | | | |
| --- | --- | --- | --- | --- | --- | --- | --- | --- |
|  | Own production | Received from food for work | Purchased from market | Received from remittance/gift | Received From food aid or relief | Amount sold | Seed reserves | post-harvest loss |
| Maize |  |  |  |  |  |  |  |  |
| Barley |  |  |  |  |  |  |  |  |
| Wheat |  |  |  |  |  |  |  |  |
| Rice |  |  |  |  |  |  |  |  |
| Tef |  |  |  |  |  |  |  |  |
| Sorghum |  |  |  |  |  |  |  |  |
| Sweet potatoes |  |  |  |  |  |  |  |  |
| Potatoes |  |  |  |  |  |  |  |  |
| Cabbage |  |  |  |  |  |  |  |  |
| Carrot and related |  |  |  |  |  |  |  |  |
| Bean |  |  |  |  |  |  |  |  |
| Pea |  |  |  |  |  |  |  |  |
| Lentils |  |  |  |  |  |  |  |  |
| Chickpeas |  |  |  |  |  |  |  |  |
| Avocado |  |  |  |  |  |  |  |  |
| Orange |  |  |  |  |  |  |  |  |
| Apple |  |  |  |  |  |  |  |  |
| Papaya |  |  |  |  |  |  |  |  |
| Banana |  |  |  |  |  |  |  |  |
| Tangerine |  |  |  |  |  |  |  |  |
| Mango |  |  |  |  |  |  |  |  |
| Meat |  |  |  |  |  |  |  |  |
| Egg |  |  |  |  |  |  |  |  |
| Fish |  |  |  |  |  |  |  |  |
| Milk |  |  |  |  |  |  |  |  |
| Sugarcane |  |  |  |  |  |  |  |  |
| Sugar |  |  |  |  |  |  |  |  |
| Honey |  |  |  |  |  |  |  |  |
| Butter |  |  |  |  |  |  |  |  |
| Processed oil |  |  |  |  |  |  |  |  |

**Appendix 1**

**Kilocalorie Conversion Table**
